# Immune infiltration landscape and immune-marker molecular typing of pulmonary fibrosis with pulmonary hypertension

**DOI:** 10.1186/s12890-021-01758-2

**Published:** 2021-11-25

**Authors:** Haomin Cai, Hongcheng Liu

**Affiliations:** Department of Thoracic Surgery, Shanghai Pulmonary Hospital, Tongji University School of Medicine, Shanghai, China

**Keywords:** Immune infiltration analysis, Weighted correlation network analysis, Protein interaction network, Disease classification

## Abstract

**Background:**

Pulmonary arterial hypertension (PH) secondary to pulmonary fibrosis (PF) is one of the most common complications in PF patients, it causes severe disease and usually have a poor prognosis. Whether the combination of PH and PF is a unique disease phenotype is unclear. We aimed to screen the key modules associated with PH–PF immune infiltration based on WGCNA and identify the hub genes for molecular typing.

**Method:**

Using the gene expression profile GSE24988 of PF patients with or without PH from the Gene Expression Omnibus (GEO) database, we evaluated immune cell infiltration using *Cibersortx* and immune cell gene signature files. Different immune cell types were screened using the Wilcoxon test; differentially expressed genes were screened using *samr*. The molecular pathways implicated in these differential responses were identified using Gene Ontology and Kyoto Encyclopedia of Genes and Genomes functional enrichment analyses. A weighted co-expression network of the differential genes was constructed, relevant co-expression modules were identified, and relationships between modules and differential immune cell infiltration were calculated. The modules most relevant to this disease were identified using weighted correlation network analysis. From these, we constructed a co-expression network; using the *STRING* database, we integrated the values into the human protein–protein interaction network before constructing a co-expression interaction subnet, screening genes associated with immunity and unsupervised molecular typing, and analyzing the immune cell infiltration and expression of key genes in each disease type.

**Results:**

Of the 22 immune cell types from the PF GEO data, 20 different immune cell types were identified. There were 1622 differentially expressed genes (295 upregulated and 1327 downregulated). The resulting weighted co-expression network identified six co-expression modules. These were screened to identify the modules most relevant to the disease phenotype (the green module). By calculating the correlations between modules and the differentially infiltrated immune cells, extracting the green module co-expression network (46 genes), extracting 25 key genes using gene significance and module-membership thresholds, and combining these with the 10 key genes in the human protein–protein interaction network, we identified five immune cell-related marker genes that might be applied as biomarkers. Using these marker genes, we evaluated these disease samples using unsupervised clustering molecular typing.

**Conclusion:**

Our results demonstrated that all PF combined with PH samples belonged to four categories. Studies on the five key genes are required to validate their diagnostic and prognostic value.

## Background

Pulmonary fibrosis (PF) is a progressive type of interstitial lung disease that causes fibrotic destruction of the lung parenchyma caused by repeated damage to the alveolar epithelium or endothelium [1]. This damage causes fibroblasts to excessively secrete extracellular matrix components such as collagen and fibronectin, which in turn lead to lung scarring (fibrosis) [2, 3]. The occurrence of PF leads to reduced lung function and respiratory failure, and—as the disease develops—the respiratory function of the patient continues to deteriorate [3–5]. However, the exact cause of PF remains unknown.

Currently, the treatments for PF include lung transplantation or the use of anti-fibrosis drugs (pirfenidone and nintedanib), immunosuppressive agents (prednisone), and chemotherapy (cyclophosphamide) [6]. During treatment, obesity [7], diabetes [8], gastroesophageal reflux [9], pulmonary hypertension [10], obstructive sleep apnea [11], coronary artery disease [12], and emphysema [13] can lead to changes in clinical symptoms and of the condition of the patient. Pulmonary arterial hypertension (PH) secondary to PF (associated pulmonary hypertension, APH) is one of the most common complications in PF patients and is characterized by severe pulmonary vascular remodeling and capillary density reduction, leading to a continuous increase in pulmonary pressure [14, 15]. Patients with various forms of end-stage PF require lung transplantation, and the presence of PH increases the risk of these surgeries in these patients. Recently, researchers have increasingly recognized that PH is a major contributor to PF morbidity and mortality [16, 17]. Therefore, a better understanding of the key genes in APH is likely to support better molecular typing of this disease and improve our understanding of its clinical significance.

Weighted correlation network analysis (WGCNA) is a systematic biological method that can be used to identify highly correlated gene modules to identify candidate biomarkers or therapeutic targets [18, 19]. Using WGCNA, Qi et al. [20] found nine long non-coding RNAs (*UCA1*, *PVT1*, etc.) which may be related to the progression of non-small-cell lung cancer using WGCNA analysis, providing new possible molecular targets for the diagnosis and treatment of this disease. Wang et al. [21] identified four key genes associated with idiopathic pulmonary fibrosis (IPF) (*COL14A1*, *TSHZ2*, *IL1R2*, and *SLCO4A1*). In addition, the application of WGCNA in lung cancer and IPF studies also suggests that it is feasible to combine the data in the Gene Expression Omnibus (GEO) database and use WGCNA to identify key genes related to specific conditions in an effort to produce a genetic signature of the disease.

This study aimed to screen the key modules associated with APH immune infiltration and identify the hub genes for use in molecular typing. We identified the differential immune cell types associated with PF with or without APH using a single dataset downloaded from the GEO database and used these data to identify the differentially expressed genes, enriched biological pathways, and key regulator nodes for APH. We hope that our data will provide a reference for further research focusing on the classification and treatment of APH.

## Methods

### GEO data download and preprocessing

We obtained the gene expression dataset describing APH (GSE24988, published on October 28, 2010) from the GEO database. This database, last updated on the 26th of July 2018, contains data from 116 samples. These data were obtained from fresh frozen lung samples collected from the recipient organs of 116 patients with PF undergoing lung transplantation. The transcriptional profiles of these samples were obtained using an Affymetrix microarray and these 116 samples included 17 samples with severe PH (mean pulmonary arterial pressure, mPAP > 40 mmHg), 22 samples without PH (mPAP < 20 mmHg), 45 samples with intermediate PH (mPAP 21–39 mmHg), and 32 control samples.

### Immune cell infiltration analysis

The R function and 22 immune cell gene feature files provided by *Cibersortx* (http://cibersortx.stanford.edu) were used to calculate the degree of immune cell infiltration in the GSE24988 samples, and the percentage of immune cells in each sample was displayed using a stacked histogram. Next, we created a ratio matrix of these immune cells (noPH–PF: PH–PF) to identify the differences in their proportions in different sample types using the Wilcoxon test (*p* < 0.05).

### Identification of differentially expressed genes

Differences in gene expression between PH and non-PH samples in the GSE24988 dataset were evaluated using the *samr* R package, and differentially expressed genes were selected using a *p* value of < 0.05, and 1.2 as the fold-change (FC) threshold [22, 23].

### Functional enrichment analysis

Gene Ontology (GO) and Kyoto Encyclopedia of Genes and Genomes (KEGG) pathway enrichment analyses are widely used for high-throughput gene analysis. The *Metascape* database provides a comprehensive set of functional annotation tools to understand the biological significance of many genes. In this study, the *Metascape* database was used to perform GO biological process and KEGG pathway enrichment analyses of the differentially expressed genes identified in the GSE24988 dataset.

### Construction of a co-expression network

The WGCNA package in R was used to construct a weighted co-expression network for differentially expressed genes in the GSE24988 dataset. The coefficient of variation for each gene was calculated using WGCNA analysis, and a weighted gene co-expression network was constructed. Finally, we selected an appropriate module size to identify the co-expression modules.

### Identification of the key modules

We calculated the correlation between gene–immune cell significance and the degree of connectivity within each module and then screened out the module most relevant to the immune cell phenotype based on the gene significance (GS) and module-membership (MM) thresholds.

### Screening of the hub genes associated with differences in immune cell infiltration

The *STRING* database can be used to predict protein–protein interactions (PPIs), which can then be used to retrieve protein–gene interactions verified by experiments or predicted based on the literature. We extracted and identified the genes in the key modules, constructed a PPI network using the *STRING* database, and then extracted the hub genes from this network. Once this was completed, we identified the key module hub genes (according to MM and GS) and selected the threshold of gene significance (geneTraitSignificance) as needed. Here, we used the official website’s default value of 0.2; MM was set to 0.8, and the key module was obtained after screening the gene. Finally, the key genes in each module and the key hub genes from the PPI were combined to produce a panel of key marker genes associated with immune cell infiltration in APH.

## Results

**Fig. 1:**
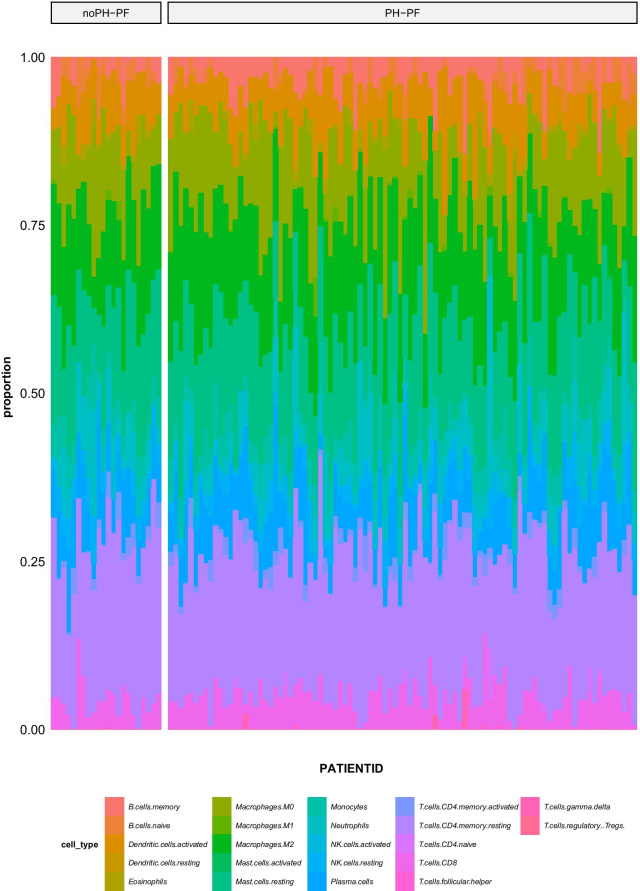
Stacked histogram of 22 types of immune cell infiltration corresponding to the dataset

To obtain the clinical sample data for APH, we downloaded the APH hypertension chip data within GSE24988 from the GEO database. This dataset contains results from 116 samples. We first matched the probe data from the chip with the gene data and excluded any data that did not have a corresponding gene name or which were duplicates. We then averaged the remaining gene expression values to obtain the final sample values and named them 22noPH–PF and 94PH–PF. The R function and 22 immune cell gene signature files provided by *Cibersortx* were used to calculate the degree of immune cell infiltration in each sample. The stacked histogram shows the proportion of immune cells in each sample (Fig. 1).

**Fig. 2:**
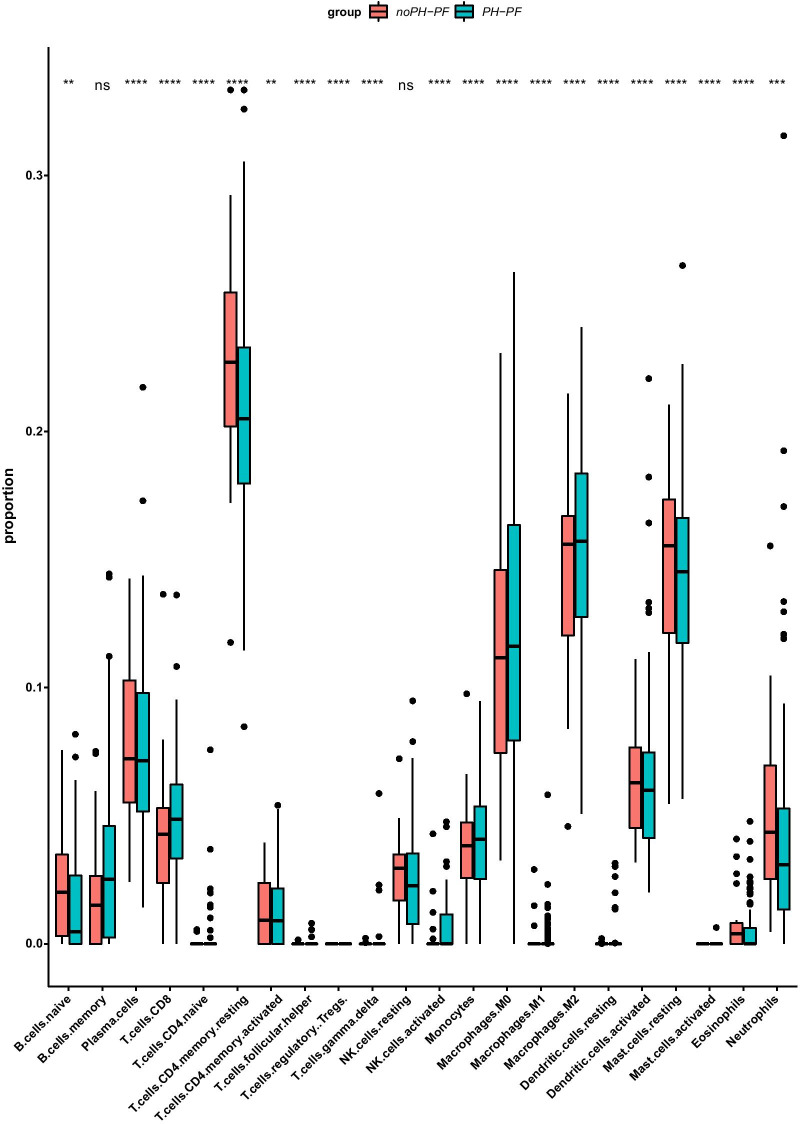
Immune cell difference analysis box plot

To study the proportion of the same type of immune cells in different samples, we used the ratio matrix (noPH–PF: PH–PF) and evaluated the differences in the abovementioned immune cells in each sample using the Wilcoxon test (*p* < 0.05). The results showed that in addition to memory B cells and resting NK cells, the remaining immune cells were significantly different between the noPH–PF and PH–PF groups (Fig. 2). This suggests there is a certain degree of immune overlap between the noPH–PF and PH–PF patients.

### Screening for differences in noPH–PF and PH–PF gene expression profiles

**Fig. 3:**
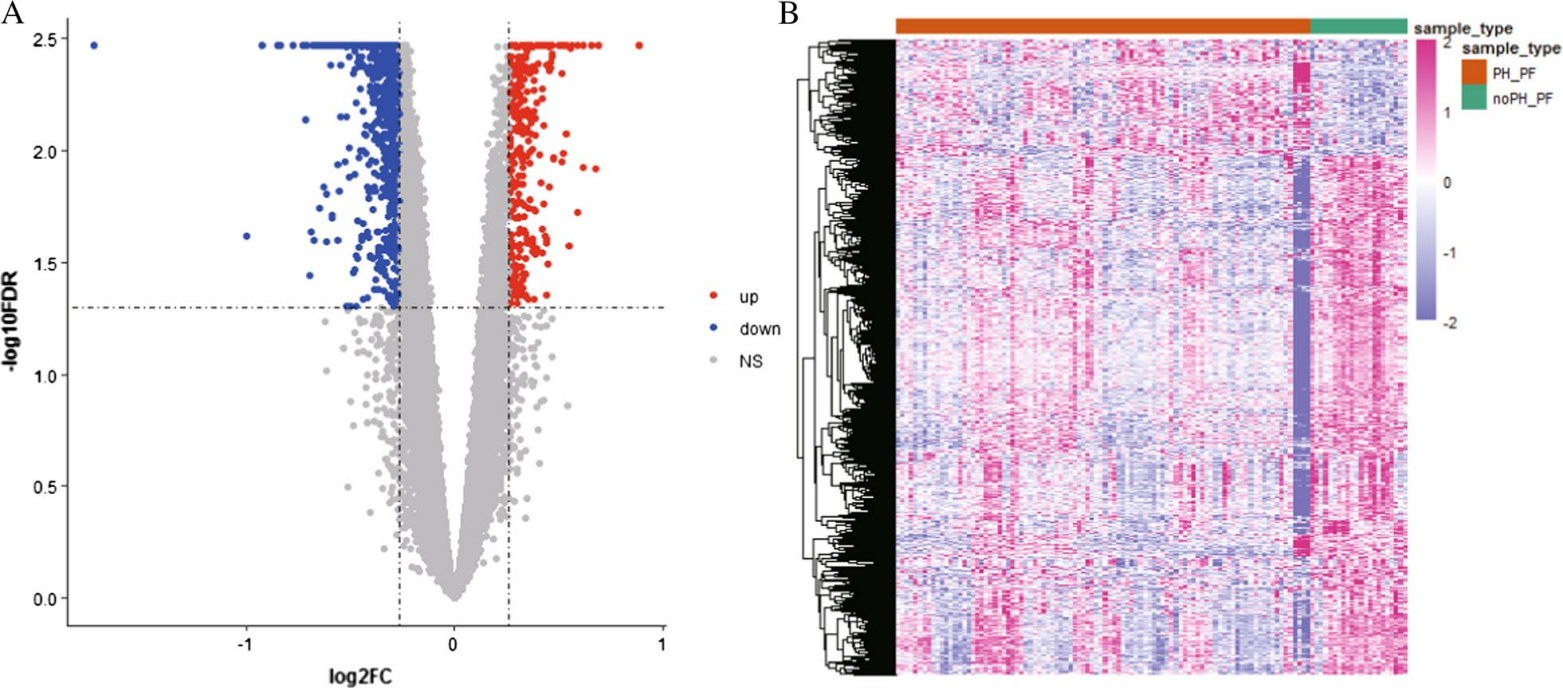
Differential gene expression analysis. **A** Differential gene volcano map. **B** Heat map of differential gene expression

To identify the differentially expressed genes associated with APH and PF, we used *samr* in R to screen for the differential genes and select those with a corrected *p* value of < 0.05 and FC of 1.2. This analysis identified 1622 differentially expressed genes, of which 295 were upregulated and 1327 were downregulated (Fig. 3).

### GO and KEGG analysis of these differentially expressed genes

**Fig. 4:**
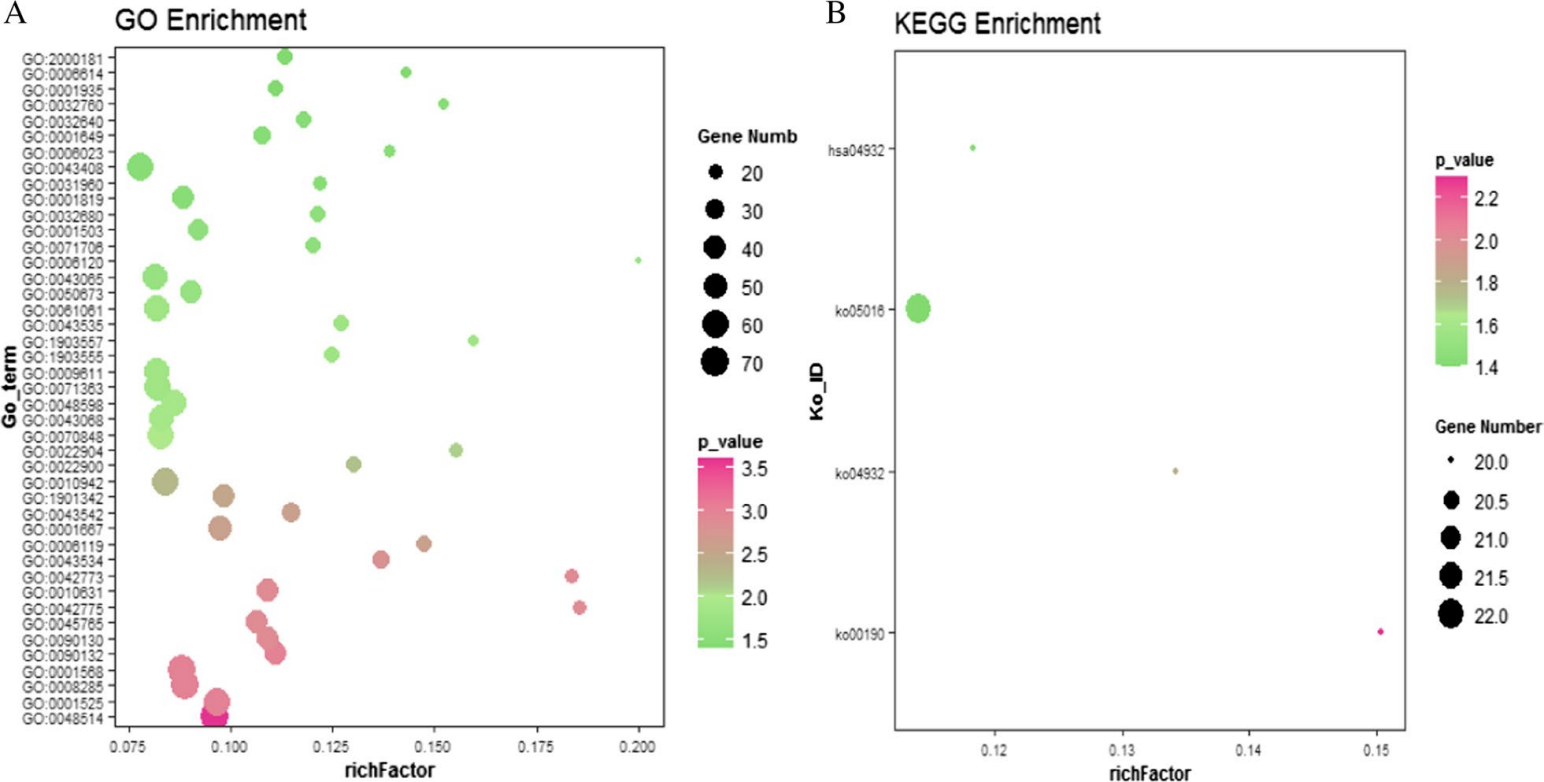
Enrichment analysis of differentially expressed GO and KEGG pathways. **A** Bubble chart of GO term enrichment of differentially expressed genes. **B** Enriched bubble chart of the KEGG pathway

To analyze the functional enrichment associated with these differentially expressed genes, 1622 differential genes were imported into the *Metascape* database and subjected to both GO biological process and KEGG analysis. Of these genes, 98.3% were enriched in signaling pathways. This result suggests that the differences between these groups are likely to be closely associated with cell-to-cell signaling and activation. The majority of the differentially expressed genes were associated with pathways such as MAPK kinase regulation and positive regulation of cell death (Fig. 4, pathway information is indicated by ID).

### Construction of the co-expression network

**Fig. 5:**
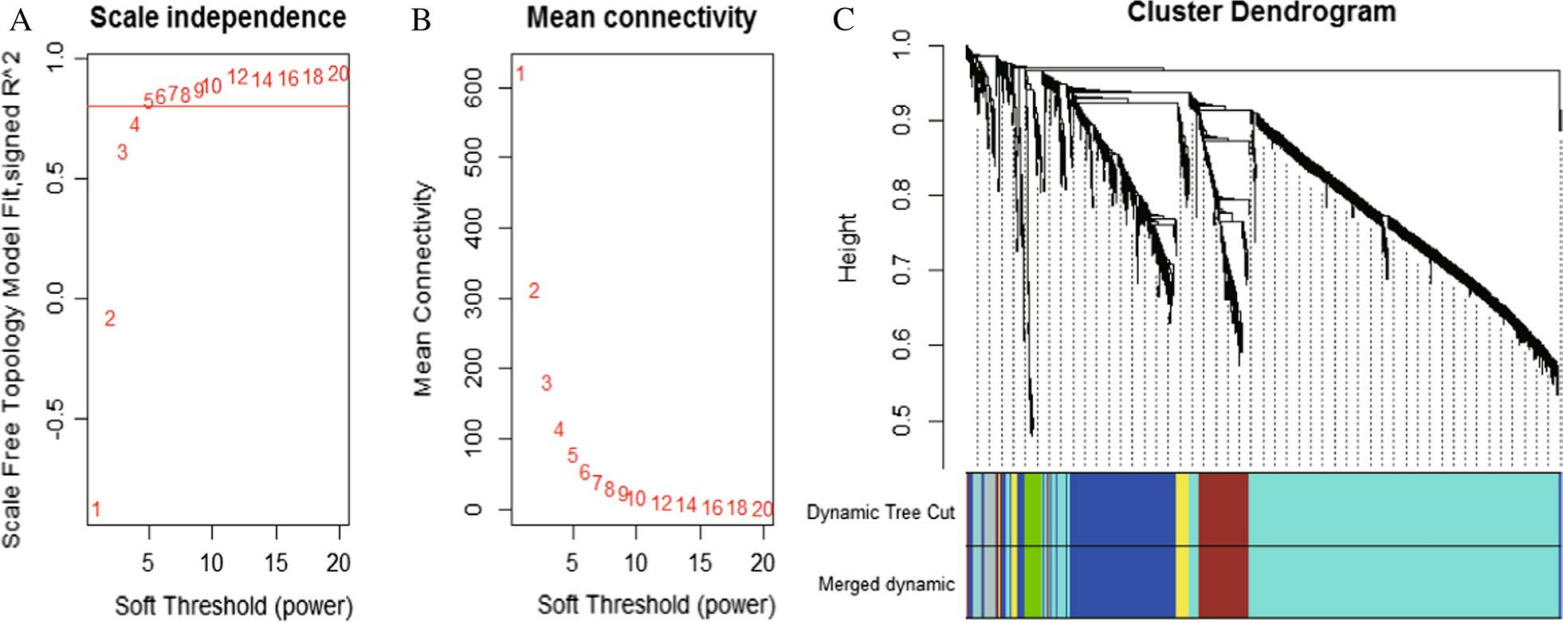
Co-expression network construction. **A**, **B** Analysis of network topology for various soft-thresholding powers in scale independence and mean connectivity. Therefore, when the soft threshold power was set to 5, the co-expression network fits a scale-free distribution. **C** Gene dendrogram and module colors

To classify and analyze the impact of the differential gene expression profiles in these tissues, we constructed a weighted co-expression network in R using the WGCNA software package. The results are shown in Fig. 5A, B (power = 5), which revealed that six modules could be identified within this data (Fig. 5C). The module size ranged from 27 to 969 genes, and the statistics underlying the number of genes in each module are summarized in Table 1.

**Table 1:** Summary of genes in each module after weighted correlation network analysis

Module	Number of genes
Blue	366
Brown	156
Green	46
Grey	27
Turquoise	969
Yellow	58

### Identification of the key modules associated with immune infiltration

**Fig. 6:**
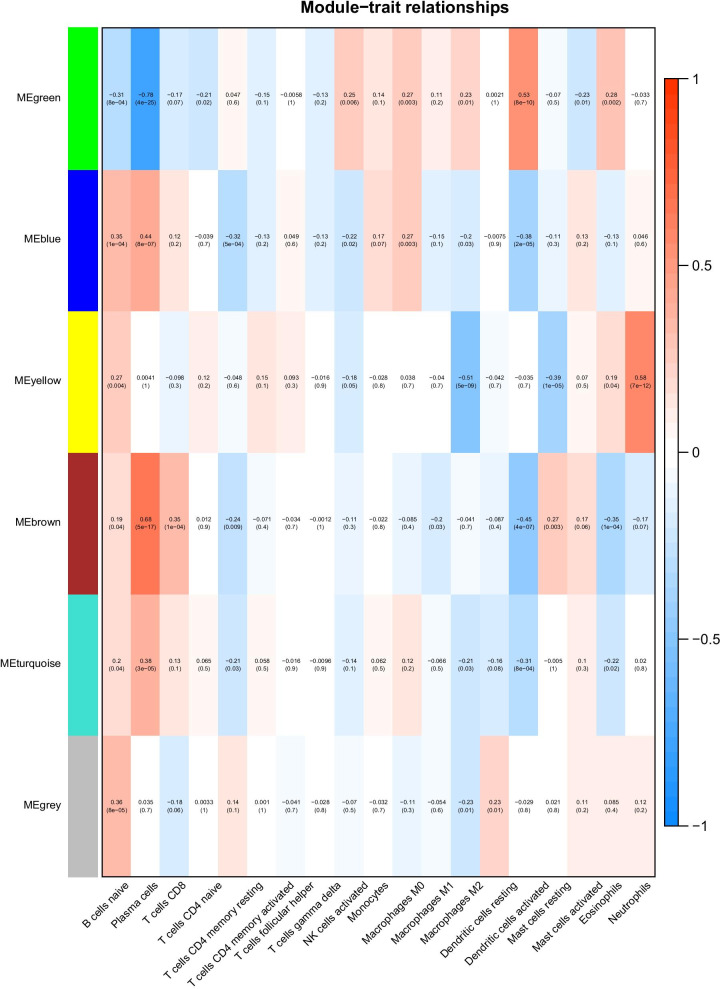
A heat map of the correlation between the module and the differential immune cells

**Fig. 7:**
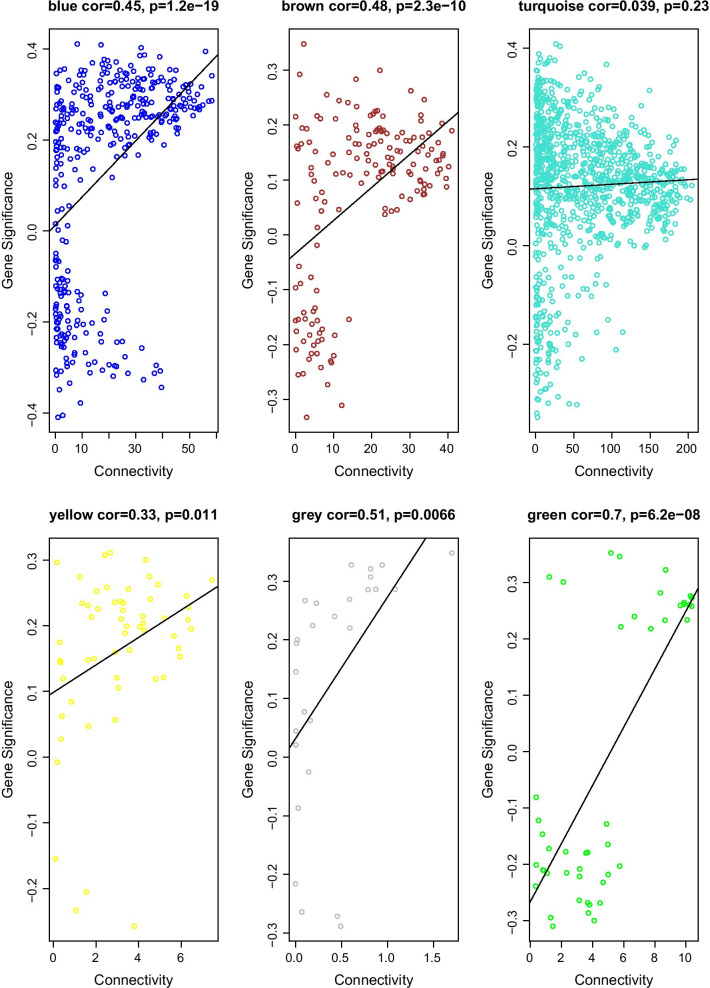
Relationship between gene–immune cell saliency and connectivity within the module

To explore the correlation between the six modules and immune cell infiltration, immune cells with significant differences in abundance between the two sets of samples were selected and used to draw a module–immune cell correlation heat map (Fig. 6). We then analyzed the correlation between the different modules and immune cell invasion. These values were then used to estimate the relationship between gene–immune cell saliency and connectivity within each module, and this parameter was then used to identify the module with the most significant impact on immune cell infiltration. Using all these data, we were able to identify the green module as being most likely to influence immune cell infiltration (Fig. 7).

### Identification of the hub genes associated with immune cell infiltration

**Fig. 8:**
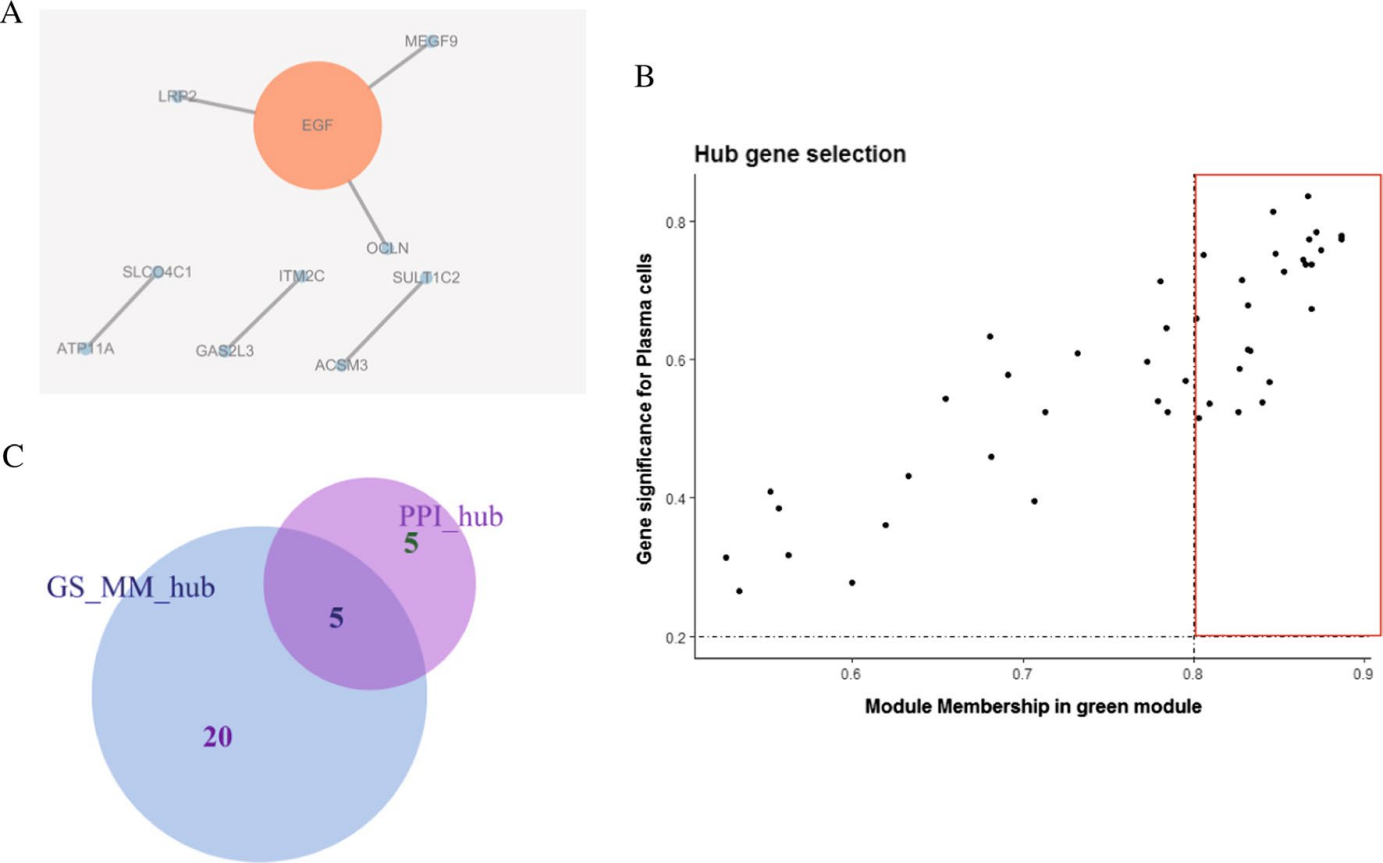
Screening of hub genes related to immune cell infiltration. **A** The protein–protein interaction network constructed using the genes from the green module in the *STRING* database. The greater the redness of the point, the larger the size, indicating that the node has more genes. **B** Identification of the key genes of the green module. The hub gene is indicated by the red box. **C** Intersection of hub genes identified from the green module and key nodes in PPI network to produce a single set of 5 critical genes associated with immune cell infiltration in APH

To further identify genes specifically associated with immune cell infiltration in the green module, we extracted the genes from the green module (46 genes in total) and constructed a protein–protein interaction (PPI) network using *STRING*. The results of this analysis revealed that there were 10 nodes in the network (not counting solitary points; Fig. 8A). We then extracted the key genes from each node in the PPI network and considered them *hub genes*. We also identified the critical genes in the key module using MM and GS scores. These evaluations identified 25 hub genes (GS_MM_hub; Fig. 8B). Finally, we investigated the intersection of the hub genes (GS_MM_hub) identified from the key module and key PPI nodes to produce a single set of critical genes (5) associated with immune cell infiltration in APH. These genes included *ATP11A*, *ITM2C*, *OCLN*, *SLCO4C1*, and *MEGF9* (Fig. 8C).

### Unsupervised clustering facilitated molecular typing of diseases based on hub gene expression

**Fig. 9:**
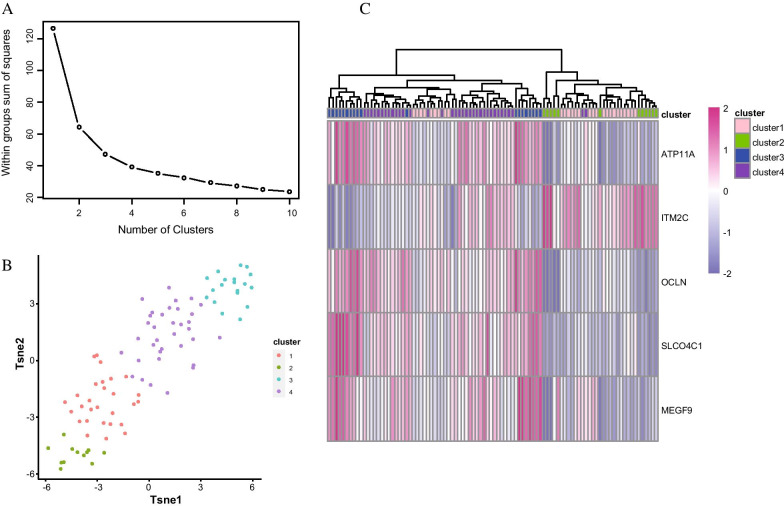
**A** The threshold is based on the sum of the squared error. **B** TSNE diagram shows the clustering of PF-PH samples. **C** The expression of five key genes in PF-PH samples

To further determine whether the obtained hub genes could be used for the molecular classification of these diseases, we mapped the five key hub genes obtained from the GSE24988 data using unsupervised clustering. We first classified all PF-PH samples using the *k*-means unsupervised clustering method and then used the inflection point of the sum of the squared error to select the optimal *k* value. As shown in Fig. 9A, the decline slows down after *k* = 4; therefore, we selected *k* = 4 as the threshold. We then used the *Rtsne* R module to reduce the dimensionality of the gene expression data, and our results showed that all PF_PH samples could be clearly divided into four categories (Fig. 9B). By combining the expression of key genes co-expressed in all disease samples (Fig. 9C), we found that no classification difference could be seen from the expression of a single gene, but a combination of all five critical genes yielded a highly consistent stratification (Fig. 9B).

### Analysis of different types of immune infiltration

**Fig. 10:**
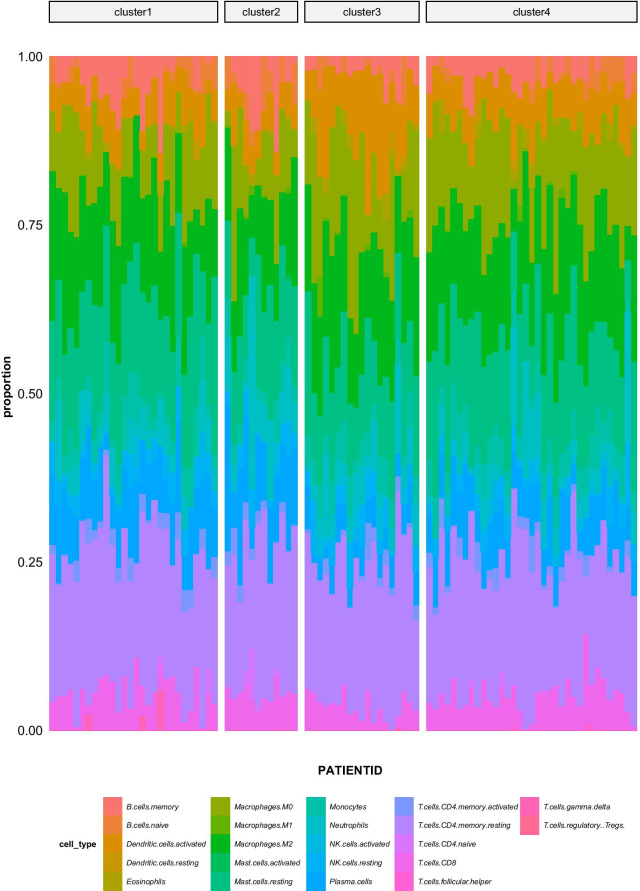
Analysis of different types of immune infiltration

To compare the differences in immune infiltration among the four types identified in the hub gene screening we again used the R function and 22 immune cell gene signature files provided by the *Cibersortx* website to calculate the differences in the degrees of immune cell infiltration among these four groups. The results of this analysis revealed that each of these four groups corresponded to a specific subset of immune cells with different characteristics, which suggests that these four groups can be used to facilitate molecular classification of these samples (Fig. 10).

### Differences in the expression of hub genes of different types

**Fig. 11:**
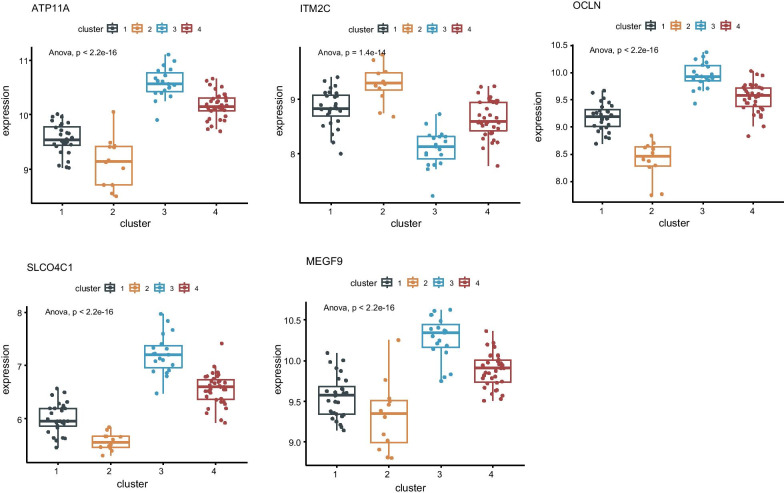
Differences in the expression of hub genes in different types

To explore the expression differences among the hub genes of different types, we calculated the expressions of each of the five hub genes in each of the four different groups and used box plots to show the differences for each gene in each group. We then used ANOVA to calculate the differences in the expression levels of these five genes among the four different types. The results showed that the expression of these genes was significantly distinct for each type (Fig. 11), suggesting that these five genes may be applicable as biomarkers for each of these four subgroups.

## Discussion

PF is a progressive and epidemic disease that primarily manifests as coughing, progressive exertional dyspnea, and restricted movement. Studies have shown that the incidence and prevalence of PF are increasing every year [24]. Many interstitial lung diseases cause diffuse pulmonary fibrosis, indicating that there are many different causes of PF. Therefore, the treatment of PF cannot be generalized. In addition, it is important to identify and accurately treat other diseases associated with PF. Of these, PH is among the most common PF comorbidities and is more likely to cause severe breathing difficulties and poor prognosis [13, 15, 25]. However, there is still a lack of accurate biomarkers for the diagnosis and treatment of PF combined with PH.

Our study used bioinformatics methods to analyze and screen several differentially expressed genes between patients with PF and PH and with PF alone from a dataset based on their general gene expression profile. Furthermore, a weighted co-expression network was constructed using WGCNA and used to identify six critical gene modules for APH. Further evaluation allowed us to identify a single critical module determining immune cell infiltration in these samples, from which we were able to identify a panel of five critical genes that may act as biomarkers for APH. This panel consisted of *ATP11A*, *ITM2C*, *OCLN*, *SLCO4C1*, and *MEGF9*. When we combined their gene expression profiles we were able to clearly identify four different categories of PH–PF and noPH–PF samples, allowing for clear stratification and potentially simplifying intervention.

The ATPase phospholipid transporting 11A (*ATP11A*) gene encodes a membrane ATPase, ABCA1, which acts as a transmembrane protein with a transport function [26]. Studies have shown that mutations in another transporter gene, *ABCA3*, can affect the function of surfactant proteins; this leads to a lack of surfactant in newborns and is therefore closely associated with desquamative interstitial pneumonia in children [27]. Quantitative transcriptomic analysis using RNA-seq showed that *ATP11A* is expressed in multiple tissues and is significantly enriched in the lungs [28]. Tasha et al. conducted a genome-wide association analysis of 1616 pulmonary fibrosis cases and 4683 control cases, and identified seven new susceptibility loci for pulmonary fibrosis, including *FAM13A*, *DSP*, *OBFC1*, *ATP11A*, *DPP9*, and chromosome regions 7q22 and 15q14–15 [29]. This supports our observation that *ATP11A* is a key gene in differential immune cell infiltration in patients with APH or PH alone, suggesting that *ATP11A* may act as a susceptibility gene for pulmonary fibrosis and an indicator of pulmonary hypertension.

The integral membrane protein 2C (*ITM2C*) gene, also known as *BRI3*, is a member of the BRI gene family. Mutations in the *BRI2* gene have been linked to the development of familial dementia in the United Kingdom (a stop-codon mutation in the BRI gene) and in Denmark (a decamer duplication in the 3′ region of the BRI gene produces an amyloid peptide associated with dementia) [30, 31]. These familial dementias are primarily associated with changes in amyloid precursor protein (APP) processing speed and the production of Aβ42 resulting from APP and presenilin mutations [32]. Shuji Matsuda et al. identified BRI3 as an interaction partner for APP. Researchers have found that overexpression of *BRI3* reduces the cleavage of APP by α- and β-secretase and increases the levels of sAPPβ, Aβ40, and Aβ42. These results indicate that BRI3 may promote the onset of Alzheimer’s disease by regulating APP processing [33]. Subsequent studies have found that BRI2 and BRI3 could be substrates for ubiquitin ligase nuclear receptor binding protein 1, which would ubiquitinate these proteins and schedule them for degradation. This means that this protein may also be a potential therapeutic target for Alzheimer’s disease [34]. To date, most studies on BRI3 have focused on its role in Alzheimer's disease; our study is the first to link BRI3 to the molecular typing of pulmonary fibrosis with pulmonary hypertension, which may provide a new avenue for BRI3 functional research.

Occludin (OCLN) was one of the first tight junction proteins to be discovered and is necessary for maintaining the structure and function of tight junctions. Wang et al. found that *OCLN* is highly expressed in multiple lung cancer tissue samples, and its knockdown can inhibit the proliferation of lung cancer cells and induce an increase in cell apoptosis, proving that OCLN is a potential therapeutic target for lung cancer [35]. Zou et al. found that the expression of *OCLN* in lung cells near the fibroblast foci of patients with pulmonary fibrosis was upregulated. The authors speculated that the increased expression of *OCLN* may indicate a tightening of the epithelial barrier, which may be an adaptive mechanism in response to pulmonary fibrosis [36]. These findings indicate the important role of OCLN in lung diseases, and whether tight junctions are changed is also an important sign of the onset or progression of lung diseases. Our research showed that OCLN plays an important role in APH. This not only indicates that *OCLN* can be used as a possible molecular classification standard for APH, but also suggests that tight junction changes may also be a new diagnostic criterion for this disease.

The solute carrier organic anion transporter family member 4C1 (*SLCO4C1*) gene is a member of the organic anion transporter family and is primarily expressed in the kidneys, lungs, liver, and other tissues. In addition to its function in chronic kidney disease, *SLCO4C1* has also been found to be involved in the occurrence of various tumors [37]. Rafael et al. found that *SLCO4C1* acts as a tumor suppressor gene for primary head and neck squamous cell carcinoma, and promoter methylation or somatic mutations in this gene may induce or augment the occurrence and development of head and neck cancer [38]. The Cancer Genome Atlas data showed that upregulation of *SLCO4C1* is closely related to the clinical stage and survival time of endometrial cancer. Researchers have found that *SLCO4C1* regulates the proliferation, apoptosis, migration, and other characteristics of endometrial cancer cells by regulating the PI3K/AKT signaling pathway [39]. By analyzing data from the GEO database, Li et al. found that methylation of the three sites within the *SLCO4C1* promoter is related to the proliferation and differentiation of prostate cancer cells, which may be a potential molecular marker and provide a reference for the prognosis of prostate cancer patients [40]. Therefore, although *SLCO4C1* is specifically expressed in the kidney, it can also perform important functions in multiple tissue diseases. Our study found that *SLCO4C1* could be a potential molecular marker for the classification of APH, and it may also be a potential therapeutic target for APH.

Multiple EGF-like domains 9 (MEGF9) are transmembrane proteins with multiple EGF-like repeats. As multiple proteins containing EGF-like repeats are believed to be involved in the development of the nervous system, it is unsurprising that some researchers have observed the expression of *MEGF9* in the peripheral nervous system [41]. Some of these results suggest that *MEGF9* is highly expressed in the sensory ganglia and related peripheral nerves [42]. Other studies have shown that MEGF9 may be a guiding molecule in the process of neurodevelopment and maturation of the nervous system. In previous studies, researchers found that *MEGF9* expression was increased in the tumor tissues of patients with multiple breast tumors, but its expression was not detected in normal breast tissues. In addition, studies have revealed that *MEGF9* may be regulated by miR-125b, which provides a new potential molecular target for the treatment of breast cancer [43]. Our study is the first to describe *MEGF9* expression in APH and provides a novel direction for further research on *MEGF9*.

## Conclusions

Both *ATP11A* and *OCLN* were identified as critical factors in APH, which is consistent with the available data on both these genes. This further supports the accuracy of our WGCNA-based approach and suggests that our newly identified key genes—including *ITM2C*, *SLCO4C1*, and *MEGF9*—may facilitate the molecular classification and diagnosis of APH. In addition to the five key genes, our study also identified multiple pathways associated with APH, providing new ideas for follow-up studies designed to evaluate the mechanism of APH and identify potential treatment options.

## Data Availability

Data analyzed in this manuscript are publicly available from the Gene Expression Omnibus (https://www.ncbi.nlm.nih.gov/geo/), accession number GSE24988.

## Abbreviations

PH: Pulmonary hypertension
PF: Pulmonary fibrosis
WGCNA: Weighted correlation network analysis
IPF: Idiopathic pulmonary fibrosis
GEO: Gene Expression Omnibus
FC: Fold-change
GO: Gene Ontology
KEGG: Kyoto Encyclopedia of Genes and Genomes
PPI: Protein–protein interaction
APP: Amyloid precursor protein
APH: Associated pulmonary hypertension
mPAP: Mean pulmonary arterial pressure
